# Thoracic Empyema Secondary to Congenital Chylothorax in a 14-Month-Old Boy with Noonan Syndrome

**DOI:** 10.1155/2021/6620353

**Published:** 2021-04-29

**Authors:** Takeru Oikawa, Chiharu Ota, Shinya Iwasawa, Takehiko Onoki, Hideyuki Ikeda, Takushi Hanita

**Affiliations:** Department of Pediatrics, Tohoku University Hospital, 1-1,Seiryo-Cho,Aoba-Ku, Sendai 980-8574, Japan

## Abstract

Thoracic empyema usually occurs as a complication of bacterial pneumonia, but in rare cases, it is caused by hematogenous dissemination secondary to nonpulmonary diseases. Congenital chylothorax or chylothorax in children is associated with maldevelopment of the lymphatic system, nonimmune hydrops fetalis, several syndromes including Down syndrome, Noonan syndrome, or Turner syndrome, a complication of thoracic surgery, right heart failure with high central venous pressure, or tumors. There are very few reports of empyema associated with preexisting chylothorax. In the present study, we describe a rare case of thoracic empyema associated with congenital chylothorax and supravalvular pulmonary stenosis associated with clinically diagnosed Noonan syndrome. It is necessary to closely monitor patients with chylothorax because they are at risk of developing severe lung infections, such as pleural empyema or lung abscesses.

## 1. Introduction

Thoracic empyema occurs in 3.5–12.5% of paediatric patients with bacterial pneumonia [1, 2]. Aerobic Gram-positive cocci, including *Streptococcus pneumoniae*, are common causative microorganisms of thoracic empyema associated with community-acquired pneumonia [3]. On the other hand, rare causes of empyema include trauma, surgery, mediastinitis, expansion of an intra-abdominal abscess [4], or hepatic hydrothorax caused by liver cirrhosis [5]. Such non-pneumonia-associated cases of empyema are due to hematogenous dissemination from nonpulmonary foci or lymphatic flow with Gram-negative bacilli such as *Escherichia coli, Klebsiella* species, or *Serratia* species [6]. Chylothorax is usually a complication of trauma or surgery, such as esophagectomy or repair of congenital heart diseases [7]. Congenital chylothorax (CC) or chylothorax in children is caused by maldevelopment of the lymphatic system, nonimmune hydrops fetalis, several syndromes including Down syndrome, Noonan syndrome (NS), or Turner syndrome, complication of thoracic surgery, right heart failure with high central venous pressure, or tumors [8, 9]. Reported cases of empyema associated with preexisting chylothorax are scarce [10, 11]. In the present report, we describe a rare case of paediatric empyema associated with CC based on Noonan syndrome.

## 2. Case Presentation

**Figure 1:**
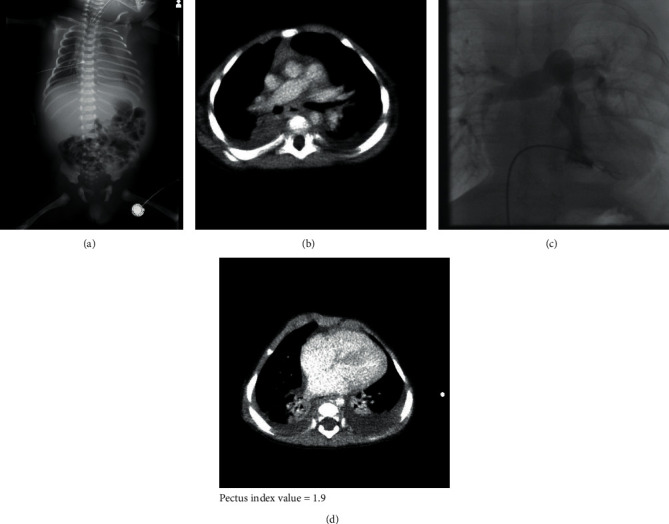
(a) Chest radiograph after birth. (b) Chest computed tomography (CT) and (c) right ventricular angiography. The arrows show supravalvular pulmonary stenosis. (d) Pectus index value calculated using CT-based imaging.

**Figure 2:**
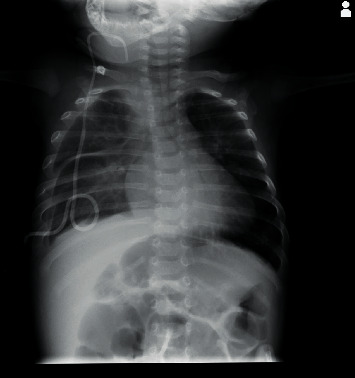
Chest radiograph at discharge from the neonatal care unit.

A 14-month-old boy presented to our department with a 2-day history of high fever and appetite loss, without any signs of upper respiratory infection such as cough or nasal discharge. He had been diagnosed with bilateral CC at 35 weeks' gestation by prenatal pleurocentesis. He was delivered by emergency caesarean section due to progressive hydrops fetalis at 36 weeks and 1 day gestation with a birthweight of 3,426 g. Immediately after birth, he was intubated in the operating room (Figure 1(a)) because of a lack of spontaneous respiration, and bilateral chest tubes were inserted. The left-sided chest tube was removed on the 13th day of life owing to gradual reduction of the left chylous fluid. He was also diagnosed with supravalvular pulmonary stenosis (PS) with a pressure gradient of 40–45 mmHg (Figures 1(b) and 1(c)) between the right ventricle and pulmonary artery. In addition to hydrops fetalis, lymphatic maldevelopment, and PS, he showed the unique facial features such as tall-forehead and low posterior hairline, hypertelorism, and down-slanting palpebral fissures, which led us to diagnose Noonan syndrome [12]. Furthermore, he showed pectus carinatum diagnosed using CT-based pectus index of 1.90 (Figure 1(d)) (diagnostic range: 1.42–1.98 [13]). Thus, according to the scoring system of clinical features [14], he had 3 major symptoms and 2 minor symptoms of Noonan syndrome (Table 1). Hydrops fetalis is also reported to be associated with Noonan syndrome [15]. Balloon angioplasty was performed on day 54 of life, which proved to be ineffective. Right-sided chylous fluid was constantly drained until around 30–40 days, and then the draining was gradually decreased and stopped at 71 days of life after various treatments, including intravenous administration of prednisolone and octreotide complemented with serum albumin and immunoglobulin and feeding with a medium-chain triglyceride (MCT) formula. Although he temporarily tolerated an increased ratio of breast and ordinary milk to the MCT formula, reincreasing the right chylous fluid led him to be fed by pure MCT formula with essential unsaturated fatty acids and fat-soluble vitamins. He was discharged on the 141st day of life from the neonatal care unit with some residual pleural effusion on the right (Figure 2).

On physical examination, he weighed 8.9 kg (1.0 standard deviation), and his height was 75 cm (0.8 standard deviation). His temperature was 39.8 C, blood pressure was 95/45 mmHg, heart rate was 180 bpm, and oxygen saturation was 98% with 1.5 L/min of oxygen inhalation through a face mask. He showed no otolaryngologic findings. On lung auscultation, right-sided breath sounds were decreased with chest retractions and tachypnoea. His heart examination revealed a grade-3 systolic murmur in the left second intercostal space. Laboratory findings indicated an increased inflammatory response with a white blood cell count of 38,200 *μ*L, 83.4% neutrophils, and C-reactive protein level of 27.49 mg/dL. Immunological studies revealed that his immunoglobulin ratio (G/M) was 975/96 mg/dL, complement component ratio (3/4) was 99/22.3 mg/dL, complement activity (CH50) was 42.3 U/mL, and the percentages of CD^4+^ T cells, CD^8+^ T cells, CD^19+^ B cells, and CD^56+^ NK cells were 21.8%, 9.0%, 41.8%, and 15.7%, respectively. Urinalysis showed no signs of urinary tract infection.

**Figure 3:**
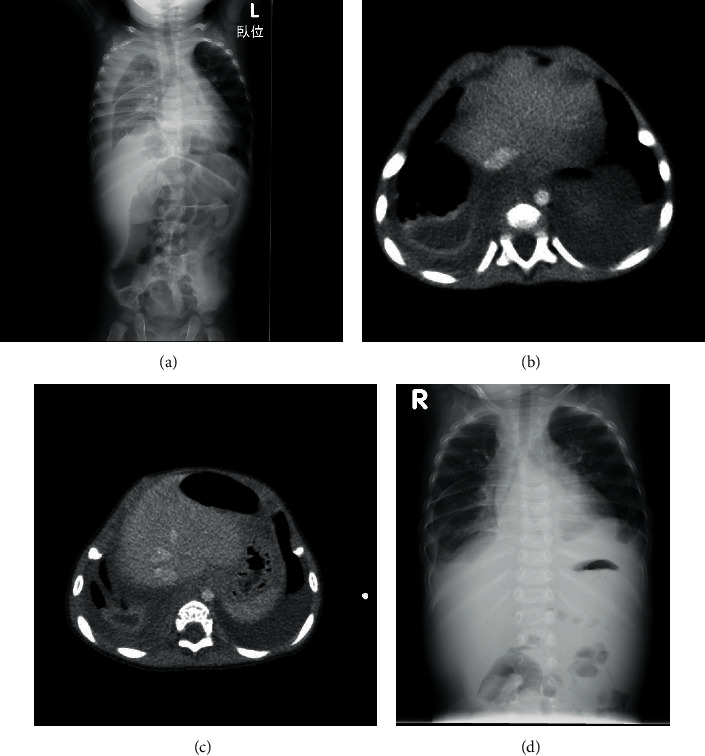
(a) Chest radiograph on admission. (b) Contrast-enhanced CT on the 18th and (c) 55th day of admission. (d) Chest radiograph at discharge.

Chest radiography revealed pleural effusions within the entire circumference of the right lung with prominent intestinal dilatation (Figure 3(a)). We percutaneously drained purulent pleural effusions and started empirical antibiotic therapy with ceftriaxone. The triglyceride concentration of pleural effusions was 299 mg/dL, and lactate dehydrogenase (LDH) level was 1214 U/mL, which confirmed the diagnosis of chylothorax [16, 17]. Two days later, *E. coli* was detected in the effusions and blood culture which were obtained on admission. In view of the sensitivity to aminobenzyl penicillin (ABPC), we decided to deescalate ceftriaxone to ABPC. Although respiratory symptoms resolved with decreased levels of inflammatory biomarkers, intermittent fever continued. On the 18th day of hospitalization, the boy developed high fever, and contrast medium-enhanced computed tomography (CT) revealed a low-density lesion (3 cm in diameter) with a high-density capsule in the right inferior lobe (Figure 3(b)). The lesion was diagnosed as a lung abscess that resulted from thoracic empyema. Medication was changed from ABPC back to ceftriaxone for another three weeks. After confirming abscess improvement on CT and chest radiograph (Figures 3(c) and 3(d)), the patient was discharged from our department on the 59th day of hospitalisation. Cardiac catheterization to evaluate PS before discharge showed 7 mmHg of right atrial pressure (RAP), systolic right ventricular pressure (RVP)/end-diastolic pressure (EDP) of 90/10 mmHg, and main pulmonary arterial pressure (PAP) of 24/10 (mean 16). Thus, RV-main PA pressure gradient was 66 mmHg. He underwent surgical repair of the supravalvular PS. Postoperative echocardiography revealed trivial tricuspid valve regurgitation (TR) with estimated RVP of 24 mmHg and PS of 1.6 m/s with estimated RV-PA pressure gradient of 10 mmHg. After the surgery, there was no recurrence of empyema or chylothorax.

## 3. Discussion

In the present report, we diagnosed the case as non-pneumonia-associated bacterial empyema caused by CC with supravalvular PS based on Noonan syndrome because respiratory failure progressed without preexisting respiratory symptoms such as otolaryngologic symptoms, rhinorrhea, or cough, and *E. coli* was detected in the patient's blood and pleural effusions obtained on admission. Noonan syndrome is a genetic disorder with mutations in the RAS-MAPK pathway, characterized by specific facial features, delayed neurological development, growth retardation, congenital heart diseases including PS, hypertrophic cardiomyopathy, or intracardiac defects such as ventricular septal defect, lymphatic maldevelopment, renal anomalies, and hematological disorders [12]. Although our patient did not undergo genetic testing, we diagnosed the patient as Noonan syndrome with the combination of facial features, cardiac disease, and lymphatic maldevelopment. Supravalvular PS increased the RVP with high central venous pressure and caused venous stasis, which may worsen CC. It has been reported that patients with high central venous pressure (CVP) after total cavopulmonary connection are at higher risk of chylothorax due to failure of the lymphatic circulation to drain excess lymph fluid back into the venous circulation [17]. In our case, the patient presented high RVP with moderate CVP and clinically diagnosed lymphatic maldevelopment due to Noonan syndrome, which might cause the continuous CC. Furthermore, although it has been indicated that the coexistence of empyema and chylothorax is rare because of the bacteriostatic nature of chyle, several reports have described cases of infected chylothorax [18, 19]. In such cases, it was speculated that the loss of activated lymphocytes in chyle [20], hepatic hydrothorax [5], or concomitant immunosuppressive therapy [21] caused immunodeficiency. It has been reported that CC increases the risk of secondary immunodeficiency caused by lymphopenia or loss of immunoglobulins and various antibodies which may cause infectious chylothorax [8]. Thus, it is possible that long-standing, continuous chylothorax would be the risk for empyema like our case.

In our patient, the route of infection can be explained in several ways. First, urinary tract or gastrointestinal infection may have been transmitted to the bloodstream and then to the pleural cavity, which led to empyema. Second, urinary tract or gastrointestinal infection may have disseminated to the pleural cavity through the lymphatic channels to cause empyema and bacteraemia. The latter hypothesis is suspected, in view of the patient's aberrant lymphatic system in addition to CC, in which pathogens proliferate more easily. In addition, the patient showed no signs of urinary tract infection, but a dilated bowel with high abdominal pressure on admission. The pressure gradient between the abdominal cavity and thorax may have caused a valve effect to mobilise intra-abdominal content to the thorax especially because the lymphatic system was compromised [8]. On the other hand, as described above, another possible course of infection is that long-standing, already infectious chylothorax might be a cause of bacteraemia in this case.

In conclusion, it is important to closely monitor patients with chylothorax, maldevelopment of lymphatic vessels, and/or increased CVP, such as those with congestive heart failure, because they are at risk of developing severe lung infections, such as pleural empyema or lung abscesses.

## Figures and Tables

**Table 1:** Diagnostic scoring of Noonan syndrome.

Feature A = major B = minor		
(1) Facial	Typical face dysmorphology	Suggestive face dysmorphology
(2) Cardiac	Pulmonary valve stenosis, hypertrophic obstructive cardiomyopathy, and/or ECG typical of NS	Other defects
(3) Height	<*P*3^*∗*^	<*P*10^*∗*^
(4) Chest wall	Pectus carinatum/excavatum	Broad thorax
(5) Family history	First degree relative with definite NS	First degree relative with suggestive NS
(6) Others	Mental retardation, cryptorchidism, and lymphatic dysplasia	One of mental retardation, cryptorchidism, and lymphatic dysplasia

Definitive NS: 1 “A” plus one other major sign or two minor signs; 1 “B” plus two major signs or three minor signs. The patient's symptoms are shown in bold. ^*∗*^*P*3 and *P*10 refer to percentile lines for height according to age, with the normal range of variation defined as *P*3–*P*97 inclusive. Referenced and modified from Table 1 of [14].

## Data Availability

The data that support the findings of this study are available from the corresponding author upon reasonable request.
